# Astrocytes and Astrocyte-Derived Extracellular Conduits in Opiate-Mediated Neurological Disorders

**DOI:** 10.3390/cells14181454

**Published:** 2025-09-17

**Authors:** Sudipta Ray, Souvik Datta, Arnab Saha, Susmita Sil

**Affiliations:** 1 Department of Pharmacology and Experimental Neuroscience, College of Medicine, University of Nebraska Medical Center, Omaha, NE 68198, USA; suray@unmc.edu (S.R.); arnab.saha.iiest@gmail.com (A.S.); 2 Department of Biochemistry and Molecular Biology, College of Medicine, University of Nebraska Medical Center, Omaha, NE 68198, USA; souvikdatta09@gmail.com

**Keywords:** astrocytes, astrocyte-derived extracellular vesicles (ADEVs), opioid-induced neuropathology, neuroinflammation, gliosis

## Abstract

Opioid-use disorder (OUD) poses a growing global health crisis, with chronic opioid exposure linked not only to addiction but also to enduring neurological impairments. While traditional research has focused primarily on neuronal alterations, emerging evidence underscores the pivotal role of astrocytes, abundant glial cells in the central nervous system, and their secreted extracellular vesicles (EVs) in opioid-mediated neuropathology. This review delineates the mechanistic roles of astrocytes and astrocyte-derived EVs (ADEVs) across a spectrum of opioids, including morphine, heroin, fentanyl, codeine, tramadol, buprenorphine, and methadone. Opioids disrupt astrocytic homeostasis by impairing glutamate regulation, altering the redox balance, and activating pro-inflammatory signaling pathways. In response, astrocytes release EVs enriched with neurotoxic cargo, including amyloidogenic proteins, cytokines, microRNAs, and long non-coding RNAs, that propagate neuroinflammation, compromise blood–brain barrier (BBB) integrity, and exacerbate synaptic dysfunction. Preclinical models and in vitro studies reveal drug-specific astrocytic responses and ADEV profiles, implicating these vesicles in modulating microglial function, neuroimmune signaling, and neuronal viability. Notably, morphine-induced ADEVs promote amyloidosis and inflammatory signaling, while heroin and fentanyl affect glutamatergic and inflammasome pathways. Even opioids used in therapy, such as buprenorphine and methadone, alter astrocyte morphology and EV cargo, particularly during neurodevelopment. Collectively, these findings advance a neuro-glial paradigm for understanding opioid-induced brain injury and highlight ADEVs as both biomarkers and mediators of neuropathology. Targeting astrocyte-EV signaling pathways represents a promising therapeutic avenue to mitigate long-term neurological consequences of opioid exposure and improve outcomes in OUD.

## 1. Introduction

Opioids are a class of potent analgesic compounds that act on the central nervous system (CNS) to aid in pain management, including pre-operative, post-operative, traumatic, and general pain-associated symptomatic care [1,2]. According to the National Institute on Drug Abuse (NIDA), opioids include prescription drugs as well as illegally procured non-prescription drugs. The common prescription opioids are morphine, fentanyl, hydrocodone, hydromorphone, methadone, buprenorphine, codeine, oxycodone, tramadol, and tapentadol [3], and non-prescription opiates include drugs like heroin, illicitly manufactured fentanyl (which is fentanyl mixed with other drugs like heroin or cocaine), as well as fentanyl analogs like carfentanil [4]. The origin of these compounds can be natural, semi-synthetic, or synthetic (Supplementary Table S1).

### 1.1. Opiates, Derivatives and Their Use

Originally derived from the opium poppy (*Papaver somniferum*), natural opiates like morphine and codeine are the oldest known analgesics used for treating severe acute pain, such as in trauma or post-surgical settings, as well as in palliative care for terminal illnesses like cancer. However, prolonged use can lead to tolerance and physical dependence, necessitating higher doses and increasing the risk of addiction [5]. Semi-synthetic opioids like oxycodone, hydrocodone, hydromorphone, and heroin, are chemically altered derivatives of the natural opiates, developed to enhance efficacy and alter absorption profiles, and are widely prescribed for moderate to severe pain, especially in chronic settings. These drugs are often formulated in combination with non-opioid analgesics like acetaminophen to enhance effectiveness. However, they are also frequently implicated in prescription drug misuse, as their euphoric effects can lead to recreational abuse and addiction [6]. Synthetic opioids like fentanyl, methadone, and tramadol, mimic the effects of opiates and are frequently included under the category due to similar activity at the opioid receptors [1,7]. Chemically synthesized synthetic opioids vary widely in potency—fentanyl, for instance, is 50 to 100 times more potent than morphine and is used in anesthesia or patients with severe opioid tolerance [8]. Others like methadone and buprenorphine, while also effective for pain, are primarily used in opioid substitution therapy due to their long half-life and ability to reduce withdrawal symptoms without producing intense euphoria [9,10]. Regardless of origin, all opiates share a common risk: the potential for dependence, addiction, and overdose. These risks stem from their action on the mu-opioid receptor (MOR), which not only modulates pain but also activates the brain’s reward system, reinforcing drug-seeking behavior [11].

### 1.2. Opiate Epidemic

Opiate-mediated neurological disorders are broadly discerned as opioid abuse disorders of the brain. However, when the drugs are used in excessive quantities over an optimal threshold in a chronic manner, it leads to drug overdoses, leading to OUDs. OUD encompasses more than 2 million individuals, with more than 100,000 deaths annually in the United States (US) [12,13]. In the US alone, opioid overdose leads to a daily toll of more than 130 deaths each day [14]. Prescription painkiller overdoses were officially declared an “epidemic” by the U.S. Centers for Disease Control and Prevention (CDC) in 2011, a turning point in public health acknowledgment of the opioid crisis. The US opioid epidemic unfolded in three distinct waves, each characterized by a change in the lead drivers of overdose-related deaths. The first, starting in the late 1990s, was caused by a rise in the prescription of opioid painkillers like oxycodone and hydrocodone, as well as methadone, triggered by aggressive pharmaceutical marketing of opioids as low risk for addiction [15,16,17], which resulted in widespread abuse and a steep increase in overdose deaths from prescription opioids. As regulations tightened, many users turned to heroin, triggering the second wave around 2010, which was marked by a high increase in deaths from heroin due to its increased availability and lower cost [18,19]. The third, starting in 2013, was a rapid rise in deaths from illicitly produced synthetic opioids, in particular fentanyl and its derivatives. These drugs are highly addictive, often combined with other opioids unknown to users, and have fueled a sharp rise in overdose death [20,21]. Some studies now warn of a potential fourth wave involving polysubstance use, such as fentanyl combined with stimulants or tranquilizers like xylazine [22]. These waves collectively highlight the evolving nature of the crisis, necessitating comprehensive public health interventions. This underscores the double-edged nature of these drugs—providing relief on one hand and contributing to public health crisis on the other [17]. While the shifting trends in opioid use underscore the need for broader interventions, the neurological consequence of chronic opioid exposure further complicates recovery, as these substances induce cognitive and behavioral impairments even beyond their addictive potential [23].

### 1.3. Opiates and Mode of Action

The effectiveness of opioids in treating acute and chronic pain has resulted in extensive medical applications, such as postoperative, cancer, and palliative care [11]. The World Health Organization’s analgesic ladder provides a stepwise approach for chronic pain management and palliative care. It advises beginning with non-opioids such as paracetamol or NSAIDs for mild pain (Step I), introducing weak opioids, like codeine, tramadol, or dihydrocodeine, for moderate pain (Step II), and advancing to strong opioids, such as morphine, oxycodone, fentanyl, or hydromorphone, for severe pain (Step III), alongside adjuvants as appropriate. Analgesic effects of opioids via binding to opioid receptors result in the activation of various signaling pathways [24]. Opioid receptors primarily fall under the G protein-coupled receptor (GPCR) family, consisting of the MOR, kappa opioid receptor (KOR), delta opioid receptor (DOR), as well as nociceptin/orphanin FQ (N/OFQ) peptide receptor [1,25,26]. Under physiological conditions, endogenous peptides like endorphins and enkephalin bind to the opioid receptors for regulating reward behavior, pain, stress, and mood, as well as breathing and gastric muscle contraction activities [27]. The exogenous drug agonists in the form of natural opiates and synthetic/ semi-synthetic opioids exert their effects by binding primarily to MORs in the central and peripheral nervous system [28]. This triggers a conformational change in the receptor, activating Gi/o-type G proteins. The Gα subunit inhibits adenylate cyclase, reducing cAMP levels and protein kinase A activity while the Gβγ subunit opens potassium channels on one hand, causing neuronal hyperpolarization decreasing their excitability, and on the other hand, closes voltage-gated calcium channels, reducing neurotransmitter release [29,30], which results in diminished pain perception, sedation, and euphoria [29,31].

Prescribing opioids remains a cornerstone of pain management, but the duration of use and the dose prescribed are key determinants of both clinical benefit and potential harm. Although the classification of short and long-term use of opioids based on duration of drug administration does not have an international standard with thresholds, it depends on approximate durations based on each opioid. Short-term opioid use refers to opioid therapy for acute or subacute pain, defined as pain lasting less than one month (acute) or one to three months (subacute), consistent with the 2022 CDC guideline [32]. Long-term opioid use refers to use on most days for longer than three months to treat chronic non-cancer pain (with outcomes examined at least 1 year later), in line with the CDC’s 2016 clinical definition [33]. According to CDC prescribing guidelines 2022, low-dose opioid therapy is generally considered 20–30 morphine milligram equivalents (MME) per day, moderate dose as 50–90 MME/day, whereas high-dose opioid therapy refers to ≥90 MME per day, with markedly increased risk of overdose at ≥100 MME/day [32]. While short-term use under medical supervision can be safe, long-term use, especially at high doses or without monitoring, can lead to MOR phosphorylation by G protein-coupled receptor kinases (GRKs). This recruits β-arrestin, which blocks further G protein signaling and promotes receptor internalization and desensitization, resulting in sustained signaling and contributing to a cycle of tolerance and dependence that fosters OUD [34,35,36,37]. The neurobiological pathways of addiction involve repeated stimulation of the mesolimbic dopamine system, reinforcing compulsive drug-seeking behavior [38]. Understanding the distinct pharmacokinetics and addiction profiles of various opiates is, therefore, crucial in tailoring safer pain management strategies while mitigating the risks of misuse and overdose. Recent approaches in pain medicine emphasize multimodal strategies that integrate pharmacological and non-pharmacological interventions, aiming to optimize pain control without over-reliance on opioids [33].

### 1.4. Physiological Consequences of Opioid Use

Chronic opiate use (>3 months) has been associated with alterations in brain structure and function, especially within the prefrontal cortex, amygdala, hippocampus, and insula—regions that govern emotional regulation, impulse control, memory, and decision-making [39]. The term ‘chronic use’ is generally used to specify opioid use on most days for more than three months, consistent with the CDC’s clinical definition of chronic opioid therapy for non-cancer pain. Functional neuroimaging studies have shown disrupted resting-state connectivity in these areas among individuals with OUD, indicative of long-term neuroplastic changes and impaired cognitive function [39]. On the other hand, prolonged use of opioids has been implicated in triggering neuroinflammatory responses through glial activation and glutamate dysregulation, as well as exacerbating excitotoxic brain injury [40]. ‘Prolonged use’ of opioids refers not to a specific temporal cutoff but rather to sustained or repeated opioid exposure for long enough period associated with addiction and drug dependence. One of the most acute risks stems from opioid-induced respiratory depression, which can cause cerebral hypoxia and irreversible brain damage if left untreated [41]. Post-mortem histopathological findings from cases of opioid overdose or intoxication psychosis reveal evidence of neural degeneration, cerebral edema, and vascular damage [42,43]. Reports have also highlighted cognitive decline and seizures in cases involving opioid-related substances such as kratom, further linking opiate toxicity with adverse neural outcomes [44]. Opiates, particularly with chronic or high-dose exposure, have been shown to compromise the integrity of the BBB, such as morphine can induce BBB permeability changes by affecting tight junction proteins, increasing oxidative stress, and activating neuroinflammatory pathways [45] or can also result in cytokine-mediated BBB disruption [45,46]. These alterations facilitate the translocation of peripheral immune cells and neurotoxic agents into the brain parenchyma, exacerbating neuroinflammation and neuronal damage.

### 1.5. Involvement of Astrocytes in Opioid-Use-Disorder

Several studies have demonstrated that opioid-led neurological disorders have been the consequence of initial opiate-triggered glial cells like astrocytes and microglia [47,48,49,50,51,52,53,54,55,56,57]. Astrocytes, the most abundant glial cells in the CNS [58], even outnumber neurons. These cells play essential roles in maintaining central nervous system homeostasis by regulating neurotransmitter and ion balance, supporting the BBB, providing metabolic and structural support to neurons, modulating synaptic transmission, facilitating waste clearance, and responding to injury and neuroinflammation [59,60,61,62,63,64]. While astrocytes are essential for neuronal survival, their dysregulation or hyperactivation in disease states can contribute to neurodegeneration [65,66]. Emerging evidence indicates that opioid-induced astrocyte loss contributes significantly to neurogenesis deficits observed in OUD [67]. Opioids, like morphine, have been shown to alter astrocyte physiology by triggering oxidative stress, disrupting glutamate metabolism, and impairing the BBB [68,69,70,71,72]. The neurological mechanism of opioid abuse disorders is elusive, involving maladaptation of immune reactivity and surveillance by glial cells through neuro-glial interactions [73]. Astrocytes modulate synaptic activity, neuroinflammation, and neurotoxic activities via the secretion of multiple conduits including neurotrophic factors, cytokines, chemokines, and EVs, with their effects depending on the specific context and the activation state of the astrocytes [74,75,76,77,78,79,80,81,82,83].

### 1.6. Role of EVs in Opioid Mediated Neurological Disorders

EVs are membrane-enclosed nanoparticles released by nearly all cell types into the extracellular space, where they facilitate intercellular communication by transferring bioactive cargo such as proteins, lipids, and nucleic acids [84,85]. EVs play significant roles in the pathophysiology of opioid-mediated neurological disorders [55,56,86,87,88,89]. There has been involvement of astrocytic conduits during neuroinflammation and reshaping the neuronal plasticity upon chronic use, that can even affect distant organs through EVs carrying nucleic acids, proteins, and lipids [67]. Furthermore, astrocytes under morphine influence show enhanced secretion of pro-inflammatory EVs, which exacerbate synaptic dysfunction and can promote amyloid pathology in the brain [90]. The release of opioid-altered extracellular vesicles containing opioid-affected cargo can have a consequential impact on cellular communication and progression to neurodegeneration [91]. Importantly, some studies demonstrate that blocking opioid receptors or modulating the EV release process can reverse these deleterious effects, highlighting the therapeutic potential in targeting glial-EV pathways in opioid-related brain injury [55,56]. However, the impact of opioids on astrocytes is less explored. ADEVs play a critical role in mediating glial–neuronal communication. In the context of opioid abuse, opioids have been shown to alter astrocyte function and EV cargo, promoting the release of ADEVs enriched in pro-inflammatory cytokines, oxidative stress markers, and regulatory RNAs [55,56,86,92]. These vesicles can contribute to neuroinflammation, synaptic dysfunction, and neuronal injury. Studying ADEVs in the context of opioid abuse is essential to understanding how opioids disrupt neuroglial interactions and may reveal novel biomarkers and therapeutic targets for mitigating opioid-induced neurotoxicity. In this review, we will elucidate the role of astrocytes and ADEVs in mediating neurological complications, mechanism(s) involved, and the role of astrocyte-derived conduits in OUD.

## 2. Natural Opioids

### 2.1. Opium: Natural Origin of Narcotic Alkaloids

Opium, a natural alkaloid extracted from the opium poppy (*Papaver somniferum*), has long been utilized for its analgesic and sedative effects. While its derivatives, such as morphine and codeine, are used medically, non-medical consumption of opium remains a persistent public health issue, particularly in parts of Asia and the Middle East. According to the United Nations Office on Drugs and Crime (UNODC) World Drug Report 2023, approximately 61.3 million people globally used opioids in 2021, a category that includes opium, heroin, and pharmaceutical opioids, with opium use being most concentrated in Southwest Asia, notably Iran and Afghanistan [93,94,95,96]. Iran, in particular, has one of the highest rates of opium use globally, with studies estimating that over 11% of the adult population has used opium at some point in their lives [97]. Chronic opium consumption is associated with a wide array of health consequences, including respiratory depression, cardiovascular disease, and an increasing body of evidence linking it to cognitive impairment and neuroinflammation. These patterns emphasize the need for greater understanding of opium’s neurological impacts, particularly as opioid-related morbidity and mortality continue to rise globally. Opium exerts profound effects on the CNS primarily through activation of MOR, which modulate pain, reward, and autonomic functions. Chronic opium use has been associated with a spectrum of neurological impairments. These include impaired neonatal neurodevelopment [98], and cognitive deficits like significant impairments in spatial learning and memory resulting from opium-induced neuronal shrinkage and structural alterations in the hippocampus [99]. Neuroimaging studies have also revealed reduced gray matter volume in brain regions involved in executive function and emotional regulation [100]. Furthermore, long-term opium consumption in adults can lead to dysregulation of microRNAs (like miR-155 and miR-187) in the peripheral blood mononuclear cells (PBMCs) and dysregulation of pro-inflammatory cytokines (e.g., TNF-α, IL-6, IL-10), detected in the serum, which have neurotoxic effects [101]. This upregulation of miR-155 and TNF-α promotes glial activation [102,103,104,105] sustained neuroinflammation [106,107] and dopaminergic neuron loss [108]. These inflammatory responses may contribute to neurodegeneration and increased risk of neurological disorders, including Parkinsonism (by modulating alpha-synuclein aggregation) [101,103]. Additionally, opium’s depressant effect on brainstem respiratory centers can lead to hypoxic brain injury, especially in overdose situations. Together, these findings underscore the neurotoxic potential of opium, highlighting the need for neuroprotective strategies in populations with chronic exposure.

Recent experimental studies have provided direct evidence that opium exposure affects astrocyte structure and function in the CNS [99,109]. In a 2023 study, Bakhshayesh et al. found that chronic opium exposure in Wistar rats (intraperitoneal injection of 30 µL, 75 µL, and 100 µL of opium tincture for 21 days) causes severe cognitive deficits, including hippocampal-dependent learning and memory deficits. Behavioral testing with the passive avoidance paradigm indicated that 75–100 µL opium-treated rats have less initial latency and more time spent in the dark chamber, reflecting memory retention deficits [109]. Histopathology of the hippocampus, and more specifically the dentate gyrus region, revealed a massive loss of the mature neuronal population and astrocytes, along with thinning of the granular layer. These morphological changes demonstrate that opium tincture produces direct neurotoxic actions on the brain circuitry of the hippocampus involving oxidative injury and glial malfunction. This study presents evidence in support of the adverse effects of opium tincture on brain areas crucial to cognitive processing [109]. Similarly, Abbasi et al. conducted a detailed evaluation on 8-week-old male Wistar rats exposed to 40 mg/kg of raw opium orally daily for one month and found pronounced structural damage in the hippocampus, including neuronal degeneration, and architectural disruption, including reactive astrocytes with hypertrophy and thickened processes, particularly in the hippocampal regions responsible for learning and memory [99]. These structural changes were accompanied by elevated oxidative stress markers, reduced antioxidant enzyme levels, and significant memory deficits in behavioral assays. Collectively, these studies demonstrate that chronic opium exposure induces alterations in astrocyte morphology and dysfunction, contributing to broader neuroinflammatory and neurodegenerative processes in the brain, leading to memory deficits, histopathological brain damage, and cognitive decline.

Although several studies have reported that chronic opium exposure induces astrocyte activation, oxidative stress, and neuroinflammation, the specific role of opium exposure on the composition or function of ADEVs remains largely unexplored. Given that ADEVs are key mediators of glial–neuronal communication and can carry inflammatory mediators, neurotoxic proteins, and regulatory RNAs, understanding their involvement in opioid-induced neuropathology is critical. Elucidating the effects of opium on ADEVs may uncover novel mechanisms by which astrocytes contribute to synaptic dysfunction and cognitive decline and could provide new therapeutic targets for mitigating the neurotoxic consequences of chronic opioid use.

### 2.2. Morphine: Natural Opioid Analgesic

Morphine is primarily used as an analgesic for the management of moderate to severe pain, but chronic exposure can lead to neurodegeneration, addiction, and molecular alterations. Morphine acts via MORs [110] and was the first isolated natural opioid from opium, and was widespread in the US as a source of addiction by the mid-1800s [111]. Women are more vulnerable to morphine mediated neurological deficits compared to men, which has been well-established in preclinical studies. Specifically, morphine has been reported to act through an additional toll-like receptor-mediated neuroinflammation in females. In addition, morphine exposure in females has been associated with increased axonal caspase-3 activation, a marker of apoptotic signaling and neuronal stress, which may impair neuronal integrity and synaptic function. Together, these mechanisms contribute to a reduction in the overall antinociceptive effect of morphine in females compared to males, suggesting the involvement of unique and sex-specific neuroimmune pathways that modulate opioid efficacy [112,113,114]. However, the legal use of morphine is limited. In 2020, it was estimated that 239.7 metric tons of morphine were used for conversion to other narcotics, and only 35.3 metric tons (11.4%) were used for the treatment of patients with cancer and in palliative care in low-income countries [115]. Whereas, in high-income countries (USA, UK, Austria, Australia, New Zealand, Canada, Japan, China, and Italy), the majority of medically used morphine accounted for 82.1%. The United States alone consumed 13.1 metric tons, while low- and middle-income nations, which comprise 82.6% of the global population, only used 16.9% of the total morphine available for pain relief [116]. Overdose statistics further contextualize morphine’s illicit use in public health, where overdose implies intentional or unintentional intake of high amount of the drug, which can profoundly depress the central nervous system including the respiratory centers in the brain. Overdose deaths result from dose of the drug high enough to cause respiratory arrest, which leads to hypoxia and ultimately death. A recent report showed that while morphine overdose deaths contributed to an overall 14.5%, about 85% of morphine overdose deaths came from illegal use of the drug [117]. Although opioids, as a class are still the leading cause of overdose deaths (107,941) in the United States in 2022 alone, morphine is not the sole contributor to opioid-overdose deaths. Intriguingly, it was found that when morphine was taken with other substances, it facilitated havoc neurodegenerative effects on human health implications. Similarly, in a recent observational study from the last two decades of findings, researchers showed a substantially steep increase in opioid distribution and use in morphine milligram equivalent (MME) per person metrics in the first half in comparison to the second half of study period in Florida, where opioid abuse was considered one of the major concerns in public health [118]. These findings underscore both the essential role of morphine in its illicit use for pain relief and subsequent overdose related deaths.

To unravel the neurological effects of morphine, several animal studies were performed. Mechanistically, in a preclinical model of mice administered with systemic morphine (10 mg/kg for 5 days), the mice developed gradual analgesic tolerance, which is associated with astrocytic activation in the midbrain [119]. The study further provided evidence of the upregulation of astrocytic glial fibrillary acidic protein (GFAP) associated with morphine tolerance, which was abrogated by glial activation inhibitors such as pentoxifylline and flavopiridol, indicating an early role of astrocytic activation in morphine tolerance [119]. Furthermore, Corkrum et al. showed that astrocytes in the nucleus accumbens express MORs that facilitated opioid stimulation through calcium elevations and glutamate release and subsequently triggered slow inward currents (SICs) in neighboring neurons, which were also shown to be inhibited by deletion of astrocytic MORs or IP3R2. These findings underscore a direct astrocyte–neuron signaling mechanism modulated by opioids [51]. Similarly, Shen et al. demonstrated that chronic morphine exposure in spinal astrocytes upregulates connexin 43 (Cx43), inducing morphine tolerance and astrocyte activation [120]. Beyond tolerance, morphine’s interaction with astrocytes has also been shown to have contrasting neurotoxic and neuroprotective effects. Skupio et al. demonstrated the involvement of astrocytic glucocorticoid receptor-based signaling associated with neuronal excitability and plasticity in the nucleus accumbens [121]. In vitro studies showed that glucocorticoid receptor activation by dexamethasone (DEX, 4 mg/kg i.p. in vivo; 100 nM for 24 h) resulted in an increased glucose uptake by ~30%, lactate release by ~25–30%, and reduced glycogen content by ~40% in primary mouse astrocytes, which were not observed in glucocorticoid receptor knockdown cells. In mice model, morphine (5–10 mg/kg, subcutaneous) administration was followed by a six-session conditioned place preference (CPP) protocol. Although control and astrocytic glucocorticoid receptor knockdown mice both developed CPP, the knockdown mice demonstrated significantly enhanced (~30–40%) preference for the morphine-paired compartment. Electrophysiological studies also revealed that the morphine-induced up-regulation in excitatory postsynaptic currents and long-term potentiation in nucleus accumbens neurons, were blocked by astrocytic glucocorticoid receptor knockdown. Systemic lactate supplementation (1 g/kg, i.p., 15 min prior to morphine conditioning) normalized this response in the knockdown mice, implying that astrocytic glucocorticoid receptor–dependent lactate release is critical for regulating morphine reward and also that morphine mediated corticosterone release disrupts normal neuron–astrocyte metabolic coupling [121]. On the other hand, in mixed rat neuronal–glial cultures, exposure to the HIV-1 envelope protein gp120BaL (CCR5-tropic, 200 pM, 24–48 h) induced ~45–50% neuronal death, whereas pretreatment with morphine (1–10 μM, 30 min–24 h) significantly reduced this toxicity by ~20–25%. This protective effect was abolished by naloxone and reproduced by the MOR agonist DAMGO (1 μM), indicating μ-receptor dependence. It was further revealed that morphine elicited a time-dependent release of the chemokine CCL5 from astrocytes, with levels rising to ~150–200% compared to baseline after 24 h, while CXCL12 release remained unaffected. Importantly, immunoneutralization of CCL5 abolished morphine’s protection, confirming that astrocyte-derived CCL5 is required for this effect. These findings demonstrate that morphine confers a neuroprotective effect against M-tropic gp120 toxicity by stimulating μ-opioid receptor–mediated CCL5 release from astrocytes [122]. Moreover, in a study by Sil et al., it was demonstrated that when rhesus macaques were administered with morphine (intramuscular) three times daily in an escalating dose of 6 mg/kg (week 1), 9 mg/kg (week 2) and 12 mg/kg for 12 weeks, which resulted in brain region-specific astrocytic amyloid generation and neuroinflammation in the frontal cortex and basal ganglia [56]. Quantitative analyses demonstrated a significant increase (~2- to 3-fold compared to saline controls) in expression of amyloid precursor protein (APP), neurotoxic Aβ1-42 AβmOC64, and the pro-inflammatory cytokine interleukin-1β (IL-1β) in the brain and a significant increase (~30-fold change) in accumulation of AβmOC64 in GFAP+ astrocytes. This mechanistic study demonstrated that Hypoxia-Inducible Factor (HIF-1α) can upregulate BACE1-mediated amyloidosis, leading to astrocyte activation and neuroinflammation. Specifically, brain-derived EVs or BEVs from morphine-administered macaques were significantly enriched in amyloidogenic proteins (e.g., APP, Aβ variants) and IL-1β. Parallelly, in vitro studies using human primary astrocytes exposed to morphine (500 nM for 0–48 h) showed similar upregulation (~3- to 4-fold compared to control) of amyloidogenic markers and inflammatory cytokines, alongside increased expression of amyloid-processing enzymes such as BACE1, as well as HIF-1α. Knockdown of HIF-1α using siRNA significantly reduced (~3- to 6-fold) morphine-induced (500 nM, 24 h) expression of BACE1, AβmOC64, and IL-1β in astrocytes. EVs isolated from morphine-treated (0.5 µM, 24 h) human primary astrocytes were enriched in HIF-1α, alongside Aβ and IL-1β. These results collectively demonstrated that HIF-1α was upregulated by morphine in astrocytes, contributing to amyloidogenic components, and was incorporated into extracellular vesicles that propagated neurotoxic signals [56]. In another study, it was found that exposure of morphine (10 μM, 24 h) on human primary astrocytes resulted in a significant ~2-fold increase in the release of ADEVs enriched with AU- and GU-rich miRNAs that activated TLR7 in microglia. Similarly, morphine-induced ADEVs from mouse primary astrocytes were found to be trafficked to endosomes in mouse primary microglia after 30 min of exposure, triggering induced NF-κB activation, with an upregulation of lincRNA-Cox2. This results in suppressing phagocytosis genes like Lrp1, Pld2, and Syk. Moreover, the knockdown of lincRNA-Cox2 restored microglial phagocytosis. Subsequently, the in vivo study on mice revealed that morphine-treatment at a dose of 10 mg/kg twice for 5 consecutive days showed ~2- to 3-fold elevated lincRNA-Cox2 levels and reduced microglial phagocytosis, while intranasal delivery of lincRNA-Cox2 siRNA-loaded EVs restored this activity, validating the therapeutic potential of targeting the TLR7–NF-κB–lincRNA-Cox2 axis [86]. Liao et al. also showed that chronic morphine treatment of starting dose of 10 mg/kg in every 8 h per day for 6 days with a daily increment of 5 mg/kg in mice led to microglial activation and elevated levels of pro-inflammatory cytokines including IL-6 (~1.5- to 2-fold) and TNF-α (~6- to 8-fold) in the thalamus which was further reversed by administration of anti-miR-138-loaded EVs. Morphine exposure (10 μM, 24 h) also increased miR-138 expression in mouse primary and human primary astrocytes, with increased packaging into ADEVs from both mouse primary (~2-fold) and human primary (~2.5-fold) astrocytes. These miR-138 packaged ADEVs from mouse primary astrocytes were taken up by mouse primary microglia (exposed to 500 EVs/cell), where miR-138 localized to endosomes activating TLR7/NF-κB signaling led to microglial activation [55]. Another study found that chronic morphine administration in mice of 3 mg/kg incremental dose leading to 12 mg/kg for 12 weeks resulted in loss of pericyte coverage at the BBB as evidenced by ~3-fold decreased NG2+/CD31+ ratios and ~3-fold decreased PDGFR-β expression, along with almost 4-fold increased monocyte infiltration in the brain, indicating BBB disruption. In addition, the study illustrated that morphine (10 μM, 24 h) induced release of ADEVs promoted downregulation of PTEN expression (~0.5-fold), leading to enhanced pericyte migration and neutrophil infiltration in human primary pericytes (after 24 h exposure to EVs) indicating that morphine-ADEVs impact BBB integrity through miRNA signaling [54]. In another study, morphine-induced ADEVs from human primary astrocytes were shown to promote primary ciliogenesis in human primary astrocytes via upregulation of miR-106b, which was inhibited by CEP97 and anti-miR106b, thus demonstrating the involvement of morphine in enhancing astrocytic ciliogenesis and drug tolerance. Postmortem brain samples from opioid users also showed ~2-fold increased primary cilia length and ~33% increased percentage of cilia on astrocytes, demonstrating clinical relevance [123]. Together, these studies reveal that morphine profoundly impacts astroglial landscapes and cargoes in ADEVs. These EV-derived signaling contribute to neuroinflammation, blood–brain barrier breakdown, amyloidosis, ciliogenesis, and impaired microglial function via specific miRNAs and lncRNAs mediated pathways, which shows both mechanistic insights and therapeutic targets for morphine-induced neuropathology.

### 2.3. Codeine: Prodrug of Morphine

Codeine is a natural derivative of morphine, which is widely used for the treatment of mild to moderate pain and cough [124]. Short-term use of codeine may lead to respiratory distress, constipation, and drowsiness [125]. The highest codeine consumption rates were reported in high-income countries, particularly in North America, Australia, and parts of Western Europe, where it is used both as a prescription and over-the-counter drug. According to the INCB, codeine stands as one of the most widely consumed opioid analgesics in north America [126]. However, misuse of codeine in combination with other substances like acetaminophen or cough syrups has been increasingly evident, leading to regulatory reclassifications in multiple countries. Although, codeine is considered less potent than hydromorphone or fentanyl, it carries a risk of misuse and overdose, particularly when combined with other CNS depressants [28].

Codeine and its analogs have increasingly been implicated in neuroinflammatory and neurodegenerative processes following chronic use [127,128,129]. In Wistar rats, prolonged oral administration of codeine-based medications (2–4 mL/kg of Archilin™ syrup and 1–2 mg/kg dihydrocodeine tablets) for 21 days led to pronounced astrogliosis in the prefrontal cortex and cerebellum, particularly at higher doses, suggesting dose-dependent glial activation [130]. In mice, chronic intraperitoneal codeine (21 mg/kg on alternate days till day 5) exposure resulted in marked hyperalgesia and allodynia with poor analgesic efficacy compared to morphine. However, activation markers like astrocytic-GFAP and microglial-CD11b, were reported to be upregulated in the spinal cord and trigeminal ganglia. These effects were dependent on TLR4 signaling and reversed by the application of IL-1R antagonists [131]. Similarly, in another Wistar rat study, 21-day codeine treatment (four groups consisting of administrating dose of 1 mg/kg, 2 mg/kg of dihydrocodeine and 2 mL/kg as well as 4 mL/kg of Archilin™ with codeine syrup, respectively) significantly reduced antioxidant defenses (SOD, CAT), increased lipid peroxidation (MDA), and downregulated neuron-specific enolase (NSE) in the prefrontal cortex and cerebellum, suggesting oxidative damage and neuronal metabolic dysfunction [132]. In summary, these results demonstrated that codeine increases oxidative stress, astrocyte activation and neurodegeneration, which warrants its careful use for a long term. Although a few reports demonstrate the role of Codeine in neuroinflammation, specific role of each glial cell type remains to be explored.

## 3. Semi-Synthetic Opioids

### 3.1. Heroin: A Semi-Synthetic Derivative of Morphine

Among the opioid derivatives available illegally, heroin emerges as a leading psychoactive agent causing morbidity and mortality worldwide. This semi-synthetic opioid heroin is derived from morphine. Heroin profoundly disrupts the CNS, leading to neuroadaptive changes associated with tolerance, dependence, and cognitive impairment. Heroin acts through MORs in the body, with increasing use in the younger population between 18 and 25 years. Reports showed that primary heroin overdose with an estimated one million at risk of developing opioid-use disorders in 2021 alone. In the United States, heroin use disorder exists among youths aged 12 years and older [133]. Recent reports state that 587,000 young individuals, who had been consuming and misusing prescription pain relievers included heroin as a major opioid in 2022 (Substance Abuse and Mental Health Services Administration, 2022) [134]. However, heroin-involved overdose deaths in the U.S. have declined, from 1.8 per 100,000 people in 2022 to 1.2 per 100,000 in 2023 [135]. Despite this decline, heroin remains a significant contributor to the overall opioid crisis.

The neurological effects of heroin are accompanied by immunological and cellular changes, particularly through EV-mediated communication that involves the central players like miRNAs and long non-coding RNAs (lncRNAs) [136,137]. Mouse and cell culture studies have provided important insights into how heroin alters astrocytic structure and function across multiple brain regions. Hynes et al. demonstrated that prolonged heroin exposure of 30 infusions of 40 μg each for 20 consecutive days in rats led to striatal reductions in astrocytic dopamine transporters (DAT) including anterior dorsolateral striatum (aDLS), involving the nucleus accumbens. Specifically, DAT expression was confirmed to be reduced significantly in heroin-treated primary astrocytes, associated with enhanced drug seeking behavior [138]. In a separate study, a 24 h heroin withdrawal in mice showed an increase in astrocyte-neuron synaptic interactions in the dentate gyrus without altering gross astrocyte morphology. Moreover, with designer receptors exclusively activated by designer drugs, the astroglial Gi-pathway was activated, which was subsequently used to explore the astrocytic function during enhanced fear learning while Specifically, astrocytes were functionally involved in mediating heroin withdrawal fear learning as significantly elevated colocalization of astrocytic membranes with neuronal post-synaptic density protein 95 was observed, indicating the neuroinflammatory role of astrocytes in post-traumatic stress disorder (PTSD) [139]. Heroin self-administration and extinction in prelimbic cortex resulted in astrocyte hypertrophy and disrupted GLT-1-dependent currents, followed by formation of and remodeled dendritic spines of PrL-NAcore projection neurons which were shown to be normalized by treatment with N-acetylcysteine [140]. Additionally, it was documented that heroin use decreased astrocyte glutamate uptake and modified inflammatory pathways, which led to synaptic dysfunction [141]. By using in vivo viral labeling in rats, investigators elucidated that heroin induced two distinct forms of astrocytic subpopulations in the nucleus accumbens core, one with enhanced morphological proximity to synapses, and other with increased extrasynaptic GLT-1 expression levels, which in turn dampen drug relapses [142]. Thus, heroin related abuse impacts through astrocytic regulation by increasing cell plasticity, synaptic dysfunction and impaired secretion of extracellular conduits like miRNA and lncRNAs. Although some studies shed light on the utility of EV mediated signaling to be associated with the neuroimmune modulation via alteration in extracellular vesicle cargo, the involvement of astrocytes and microglia is unclear [143]. The detailed pathways involving glial cells and EVs leading to astrocytic functional impairment in heroin abuse disorders are not fully comprehended and need future investigations.

### 3.2. Oxycodone: A Widely Used Painkiller

Oxycodone is a widely used semi-synthetic opioid for moderate to severe neuropathic pain and has been in clinical use since 1917 [144]. Oxycodone is clinically used in cancer-related pain management [145]. As the most prescribed drug over the years, its exploitation for illicit use led to drug abuse and its related deaths for decades [146]. Oxycodone acts by binding to both MOR and KORs [147]. Oxycodone belongs to mainstream analgesics in the US with a WHO step III opioid status along with the fentanyl drugs [148]. Oxycodone has been shown to exert effects with a lack of histamine release with higher potency than morphine [149]. Oxycodone was shown to trigger hippocampal astrocytic inactivation via NF-κB-mediated signaling in Sprague-Dawley rats [150]. A clinical study showed that along with bonafide astrocyte-derived surface markers (e.g., Glutamate aspartate transporters), there were presence of neurofilament light chain (NFL), and α-synuclein biomarkers in plasma, indicating neurodegeneration and inflammation in the cynomolgus monkeys with long term oxycodone administration of 0.001–0.17 mg/kg/injection for 3 years [151]. Using a study on female rats, oxycodone was also shown to affect maternal and offspring health negatively when taken during pregnancy for neuropathic pain [152]. Further, a reduction in astrocyte activation was associated with delayed tolerance in rats with oxycodone along with microformulations of N-palmitoylethanolamine (PEA) in the spinal cord [153]. However, studies on rats showed that extended oxycodone use triggered demyelination and neurodegeneration through apoptotic signaling pathways in the cerebellum and striatum [147]. Investigation on mice showed that oxycodone in three subcutaneous dosages of 1 mg/kg, 3 mg/kg, and 5 mg/kg for 14 days) showed reduced spinal nerve injury-induced activation of glial cells, including astrocytes, and declined pro-inflammatory cytokine levels of IL-6, IL-1β, and TNF-α, respectively, when co-administered with dexomethorphan [154]. Moreover, an in vitro study demonstrated that neuronal stem cells can undergo enhanced astrocytic or neuronal differentiation under oxycodone exposure (10 µg/mL for 48 h) [155]. Furthermore, the investigation also demonstrated that inhibiting the key regulators of ADEV release can mitigate neuroinflammatory pathways, highlighting the importance of the extravesicular axis for designing therapeutic targets [67]. Thus, astrocytes and their vesicular conduits are pivotal in mediating the neurobiological consequences of oxycodone use and present promising avenues for biomarker development, warranting further investigation.

### 3.3. Hydrocodone: A Safer Alternative to Oxycodone

Hydrocodone is an oral semi-synthetic opioid found to be a safer alternative to oxycodone in neuropathic pain. The use of the drug is associated with the risk of developing drug overdose and related deaths [156]. Hydrocodone acts by binding to MORs. Some cumulative evidence from preclinical studies demonstrated that a modifiable abuse-deterrent extended-release (ER) hydrocodone formulation could be well tolerated and equally effective for back pain [157]. A recent systematic review showed that after evaluating the relation between most opioids and their adverse events (AEs), hydrocodone belongs to a closely associated group comprising fentanyl, oxycodone, and hydromorphone opioids, sharing more than 22 adverse events through network analysis [158]. Some of the adverse events included respiratory depression, vomiting, dizziness, and constipation. Intraperitoneal hydrocodone overdose administration (40 mg/kg) was shown to trigger the enhanced astrocytic expression of mGluR5, p-nNOS/nNOS, and RAGE. The study also showed downregulation of GLT-1, xCT, as well as p-ERK/ERK expression, respectively, in mice [159]. Specifically, chronic intraperitoneal dose of 10 mg/kg for 14 days exhibited reduced expression of astrocytic glutamate transporter 1 and cysteine/glutamate antiporter in the brain regions associated with reward and downregulation of the signaling pathways involving protein kinase B (AKT), extracellular signal-regulated kinases (ERK), as well as c-Jun N-terminal Kinase (JNK) affecting synaptic function [160]. Thus, hydrocodone affects the dysregulation of the glutamatergic system of astrocytes along with triggering neuroinflammation, leading to reward-seeking behavior. Moreover, there are other glial cells that can be exploited along with astrocytes to target the glutamatergic axis in controlling neurocytotoxicity for therapeutic intervention in hydrocodone abuse and relapse.

### 3.4. Oxymorphone: A Metabolite of Oxycodone

Oxymorphone is a semi-synthetic opioid that specifically binds to MORs and is used for pain relief in various disease conditions. Unlike oxycodone, oxymorphone does not show binding affinity towards the kappa-opioid receptor [161]. Oxymorphone is a derivative of oxycodone with a relatively larger impact than oxycodone and has been in clinical use since 1959 when it was FDA approved [162]. Oxymorphone is converted by Cytochrome P450 2D6 (CYP2D6) from oxycodone and can bind to MOR with 40-fold higher affinity than oxycodone. In addition, oxymorphone sensitivity and addiction liability response have a genetic component. A seminal study on mice showed that *Zhx2* is a quantitative trait gene responsible for brain persistence of oxymorphone due to the loss of function of the transcriptional repressor *Zhx2*, which regulates the metabolism of oxymorphone. Specifically, astrocyte was shown to play a significant role in a sex-specific manner where a large intronic variant of *Zhx2* repressor-expressing gene showed reduced transcriptional expression, which subsequently leads to increased oxymorphone levels in the brain, bringing enhanced addiction behavior in females. The transcriptomics and pathway analysis study emphasized the role of astrocytes including enhanced presence of multiple astrocytic markers like ALDH1L1, GFAP, and extracellular matrix function and cell adhesion function markers in the brain, indicating the involvement of astrocytes in regulating brain addiction behavior towards oxymorphone [163]. Although there is limited literature that has shown evidence of astrocytic function in mediating oxymorphone response, it has opened a new avenue that should be directed towards finding a detailed characterization of the function of astrocytes and identifying therapeutic targets for addressing OUDs.

### 3.5. Hydromorphone: High-Potency Pain Reliever

Hydromorphone is another semi-synthetic opioid, that is a metabolite of hydrocodone [164], with greater potency over morphine [165,166,167]. Hydromorphone is approved in the US as an analgesic for moderate to severe pain [168]. Hydromorphone triggers its painkilling effect through interaction with MORs and has greater affinity for MORs than hydrocodone [169,170]. The drug is available in both short- and extended-release formulations [168]. Hydromorphone is mainly used in neuropathic pain and classified as WHO step III opioids [148]. However, in excessive quantity, hydromorphone also has the potential to be lethal [171].

Hydromorphone was first made and used in Germany in 1921, followed by its widespread use in the treatment of acute and chronic pain [172]. Since 1926, hydromorphone has been clinically used and used alternatively to morphine with a brand name Dilaudid. In a US-based study from 2007 to 2014, hydromorphone use in emergency scenarios was reported to have increased for severe neuropathic pain [173]. Further, in between 2018 and 2020, hydromorphone overdose-related deaths contributed to 681 deaths across all US with female preponderance [174]. Moreover, epidemiological evidence in Canada showed that hydromorphone caused fatality at a dose of 51 ng/mL or greater blood concentration [175]. According to the international narcotics control board annual report (2024), the hydromorphone consumption was reported to be the highest in US population [176]. Studies showed that opioid like hydromorphone, when used in anesthesia to carry out operations on ischemic insults, often reduced the astrocytic ROS generation. The study specifically showed that exclusive intravenous use of 100 nM hydromorphone demonstrated protective effect on astrocytes during surgery, indicating reduced inflammation in cerebral ischemia [177]. Hydromorphone overuse response can be mediated by astrocytic conduits and increased neuroinflammation, just like hydrocodone. The astrocytic axes can be exploited to regulate OUD due to hydromorphone using antagonists.

### 3.6. Buprenorphine: Treatment for Opioid Addiction

Buprenorphine, a partial opioid agonist, is one of the primary medications in the treatment of OUD. Buprenorphine is majorly a MOR agonist and antagonist of the kappa and delta receptors [178]. According to the International Narcotics Control Board (INCB), buprenorphine is classified under Schedule III of the 1971 Convention on Psychotropic Substances. In 2016, buprenorphine consumption data was provided to the INCB by 39 countries. Among these, Belgium recorded the highest consumption with 6.18 S-DDD per 1000 inhabitants per day, followed closely by the United States with 5.80 and the United Kingdom with 5.54. Other major consumers were Germany, Finland, Sweden, Austria, Montenegro, and Denmark [179]. In US, dispensing rates of Buprenorphine varied widely across the country, with states like West Virginia (25.9), Vermont (23.7), Kentucky (22.4), and Maine (15.7) having the highest rates per 100 persons in 2023, indicating increased consumption in eastern US than any other parts of US [180].

Buprenorphine has been shown to induce significant neurodevelopmental alterations via astrocyte-mediated mechanisms, particularly through EV signaling pathways. A pivotal study demonstrated that fetal CNS-derived extracellular vesicles (FCEs), which were extracted from maternal blood, exhibited modified molecular content upon maternal buprenorphine use [181]. These FCEs showed elevated MORs and cannabinoid receptor CB1, which indicates opioid-endocannabinoid crosstalk Interestingly, while MORs levels were increased in EVs, they were decreased in matched fetal brain synaptosomes. Further, proteomic profiling of FCEs revealed that buprenorphine exposure upregulated cytoskeletal and neurodevelopmental proteins like TUBB3 and CDC42, while downregulating key synaptic regulators such as NCAM-1 and SYT1, many of which are related to astrocyte function and synaptic stabilization. Moreover, small RNA sequencing of fetal brain tissue showed that buprenorphine significantly altered miRNA expression profiles in a sex-specific manner, with upregulation of miR-196b-5p and miR-128, which are known regulators of neuronal differentiation and astrocytic signaling. Results from a study suggested that mechanistic nexus of astrocytes and their extracellular communication channels were substantially involved in the neurobiological effects of buprenorphine exposure during pregnancy [181]. Furthermore, prenatal buprenorphine exposure did not affect excitatory synapses in neurons but significantly impaired the synaptogenic potential by astrocyte-secreted factors in an astrocyte-neuron co-culture system. In addition, brain cortical astrocytes from buprenorphine-exposed offspring showed abnormal morphology with increased lipid droplet accumulation, and altered expression of synapse-associated proteins (e.g., TSP1, TSP4, SPARC, and FABP7). These findings highlight the profound impact of prenatal buprenorphine on glial-neuronal communication during critical stages of brain development [182]. Daily prenatal administration of buprenorphine in Sprague-Dawley rats caused enduring astrocyte alterations that included oxidative stress and calcium dysregulation [183]. Similarly, in C57Bl/6J mice, oral prenatal buprenorphine disrupted astrocyte morphology, synaptogenic signaling, and lipid droplet accumulation, impairing neuron-astrocyte interactions critical for early brain development [182]. Taken together, these findings demonstrate that buprenorphine modulates glial reactivity and maturation, with important implications on the involvement of astrocytes in CNS injury and neurodevelopment. Although limited studies were found delineating the direct mechanism of astrocytes and their conduits in buprenorphine mediated disorders, research indicated a profound engagement of extracellular and EV-mediated astrocytic signaling in buprenorphine administered experiments, indicating the scope for enhanced understanding of the role of astrocytes in this drug response mechanism.

## 4. Synthetic Opioids

### 4.1. Fentanyl: Major Contributor in the Opioid Crisis

Fentanyl is a strong anesthetic cum analgesic compound that is prescribed for severe and chronic pain management. Fentanyl is one of the frontier synthetic drugs contributing to the opioid crisis in recent years [184,185]. The US contributes to the highest opioid-related overdose deaths due to the use of fentanyl [13,186,187]. Fentanyl has been illicitly overused for decades, leading to a serious public health menace [184]. Although fentanyl triggers its pharmacological effect through activation of the MORs, it has low affinity for kappa and delta opioid receptors [188]. The growing years of the 1970s distinguished the different classes of non-pharmaceutical fentanyl and fentanyl analogs to be introduced into public use [189]. Since the advent of opiate overuse in the late 1990s, synthetic opioids have created havoc on health risks in the US population. In 2022 alone, the figure for fentanyl abuse was astounding as the proportion of illicitly overdosed deaths reached more than 70% [190]. Fentanyl helps in causing reduced neuroinflammation in quick succession and acts rapidly with serious physiological effects starting from lethargy to impairment of cognitive functions, respiratory depression, arrest and coma upon overdose [191,192]. Even with a slight increase in dose, it can be lethal and act within 5 min, providing too little window period for any remedial action [193,194].

Further studies on rodents showed that repeated administration with fentanyl activates NLRP3 inflammasome in astrocytes with hyperalgesia as a signature symptom along with other common morphine- triggering symptoms like allodynia [195]. However, fentanyl drugs act primarily via µ-opioid receptors (MORs), but recent studies delineate the engagement of novel non-opioid receptors like α1-adrenoreceptors [196]. Apart from the MORs, fentanyl also targets carbonic anhydrase (CA) enzymes, which have been exploited in designing dual-targeted ligands using fentanyl templates to activate mouse glial cells for alterations in tolerance development and pain threshold [197]. Recent findings on the rat cortex indicate the absence of high-frequency astrocytic Ca^2+^ signals upon administration of fentanyl [198]. In vitro co-culture experiments suggest that not only persistent high doses of fentanyl, but even low doses of fentanyl led to neuroadaptive orchestration in synaptic transmission, remodeling of extracellular matrix, and changes in the inflammation scenario involving rodent cortical neurons, astrocytes, and oligodendrocytic cells [192]. Specifically, fentanyl activated ERK via β-arrestin-dependent pathway in mouse neural progenitor cells [199]. Fentanyl has been proposed to impact the primary rat neuron in vitro via extracellular cargo transport of miR-190 regulating the MORs [200]. Overall, in the context of opioid epidemic, fentanyl is contributing majorly due to the rapid mode of action through activation of astrocytes and their associated players.

### 4.2. Methadone: A Second Line Option for Neuropathic Pain

Methadone is a synthetic opioid analgesic and commonly used during pregnancy for pain relief. The drug preferentially binds to MORs and affects mainly the hippocampus region of the brain [201]. Especially, methadone affects the gray matter [202]. Methadone is a long-acting opioid, preferably used as cornerstone therapy in drug addiction. Methadone is attributed to unintentional overdose-related fatalities in the first decade of the millennium, when people were unaware of the effects of its overdose [203]. The analgesic is recommended as one of the two drugs, along with buprenorphine, to decrease all-cause mortality related to opiate use disorders [190]. Although methadone shows less effect than buprenorphine during use in pregnancy for lowering adverse neonatal outcomes, it has a similar effect associated with outcomes in the mothers [204]. Experiments with human cortical organoids showed delayed maturation of glial cells upon methadone exposure [205]. The chronic administration of rats indicated the substantially elevated presence of TNFα associated with induced cell death involving astrogliosis and other forms of programmed neuronal cell death [206]. Even, in the cerebellum of adult rats, methadone was found to facilitate activation of astrocytes along with microglia to trigger cell death [207]. In a post-mortem study, the reduced presence of astrocytic p-phosphoprotein (PEA-15), and phosphorylating kinase p-Akt1 was shown to be downregulated in the brains of chronic opiate addicts’ including methadone, which is further associated with altered structural and behavioral neuroplasticity and correlates of decreased total Fas associated death domain protein (FADD) as well as increased p-ser194 FADD fractions [208]. In vitro studies indicated that methadone has a direct impact on astrocytes, leading to astrocytosis through promoting pro-inflammatory signaling [209]. Overall, methadone is a second-line opioid for chronic pain relief [210]. Methadone impacts delayed maturation and astrocytosis through its activation and proves to be important in post-surgical pain relief. Overall, methadone impacts the CNS, affecting locomotor performance, motor coordination, and neuromuscular activity through dysregulation of astrogliosis along with neuronal inflammation and apoptosis.

### 4.3. Tramadol: Centrally Acting Synthetic Opioid Analgesic

Tramadol is a centrally acting synthetic analgesic used for moderate to severe pain. Approved in Germany in the late 1970s and by the U.S. FDA in 1995, it is structurally related to codeine. Its dual mechanism—weak MOR agonism and inhibition of norepinephrine and serotonin reuptake—sets it apart from conventional opioids and underlies its effectiveness in neuropathic pain, where standard opioids often fall short [211]. Due to this profile, tramadol is considered a second-line option for neuropathic pain, particularly when first-line agents like gabapentinoids or tricyclic antidepressants are ineffective or poorly tolerated [212]. Compared to stronger opioids like morphine and oxycodone, tramadol carries a lower risk of abuse and respiratory depression, supporting its widespread use in both acute and chronic pain management. It is metabolized hepatically via CYP2D6 into O-desmethyltramadol, an active metabolite that significantly contributes to its analgesic effect. This process introduces pharmacogenetic variability—ultra-rapid metabolizers, may experience enhanced effects and adverse reactions, while poor metabolizers may have reduced efficacy [213]. Though initially considered to have low dependency potential, tramadol’s long-term use has been linked to tolerance, dependence, and withdrawal, particularly with misuse or excessive dosing [214].

Between 2002 and 2013, the age-adjusted mortality rate for overdoses involving tramadol and other synthetic opioids, like fentanyl and fentanyl analogs but excluding methadone, showed minimal change, increasing from 0.4 to 1.0 per 100,000. However, a significant upward trend followed, with the rate climbing to 22.7 by 2022 [215].

Recent research highlights the impact of tramadol on the CNS beyond its simple receptor-based pain modulation. Particularly, there is growing interest in how tramadol interacts with astrocytes, glial cells crucial for maintaining CNS homeostasis, and how these interactions may involve or be mediated by EVs released by astrocytes. Tramadol, beyond its classical MOR-mediated analgesia, has been shown to induce significant glial responses, especially within astrocytic populations. In an experimental rat model, oral tramadol administration (tramadol HCL 40 mg/kg/day) led to pronounced oxidative stress, activation of the PERK apoptotic pathway, and astrocytic hypertrophy in the cerebral and cerebellar cortices. These changes were marked by the appearance of vesicular nuclei and cytoplasmic vacuolization—hallmarks of glial stress and impending apoptosis [216]. This astrocyte reactivity has been linked to neurotoxicity, a concern particularly in cases of chronic tramadol exposure or overdose. Similarly, Bekhee demonstrated that co-administration of melatonin with tramadol not only reduced GFAP expression, a marker of reactive astrocytes, but also attenuated apoptosis in cortical neurons, suggesting that astrocyte reactivity is a key contributor to tramadol-induced neurotoxicity and may be modulated through antioxidant intervention [217]. In adult male rats, tramadol exposure (tramadol HCL 50 mg/kg for three weeks) has been reported to induce significant neurotoxicity, partly mediated by astrocyte activation and oxidative stress. The hippocampus showed increased expression of GFAP, indicating reactive astrocytosis, which likely contributed to neuroinflammation and disrupted neuronal support functions [218]. Besides the adverse effects, tramadol has been shown to reduce neuropathic pain by modulating both α_2_-adrenoceptors and the activity of spinal astrocytes in the chronic constriction injury (CCI) model of the sciatic nerve in rats. Tramadol’s analgesic effect was associated with suppression of astrocyte activation, as evidenced by decreased GFAP expression in the spinal cord, which points towards the fact that tramadol not only acts through neurotransmitter pathways but also attenuates astrocyte-mediated neuroinflammation [219]. Astrocytes, therefore, play a key role in both the development and attenuation of neurodegeneration in response to tramadol treatment. In summary, tramadol’s interaction with astrocytes represents a promising yet underexplored dimension of its pharmacodynamics. These findings not only offer new insights into tramadol’s action in the CNS, but also open avenues for targeted glia-based interventions in neuropathic pain and opioid-related neurotoxicity.

### 4.4. Carfentanil: An Ultra-Potent Synthetic Opioid

Carfentanil is an ultra-potent synthetic opioid primarily developed as a veterinary anesthetic for large animals such as elephants, with a potency estimated to be 10,000 times greater than morphine and approximately 100 times more potent than fentanyl [220]. The presence of carfentanil was first noted in 2016, peaked in prevalence by 2017, and then saw a sudden and significant drop in 2018 [221]. Between January 2021 and June 2024, carfentanil was identified in 513 overdose deaths. The monthly average of such deaths rose dramatically from 3.3 per month between January 2021 and June 2023 to 34.4 per month between July 2023 and June 2024. According to the CDC data, 86.9% of the deaths involving carfentanil during the latter period also involved illegally manufactured fentanyl or its analogs. From January 2023 to June 2024, carfentanil was linked to at least one overdose fatality in 37 states, with eight states east of the Mississippi River reporting 20 or more deaths [222]. Structurally, derived from fentanyl, carfentanil binds with exceptionally high affinity to the MORs, rapidly producing profound analgesia, sedation, and respiratory depression even at microgram-level doses. Due to these extreme effects, it has no approved human use, and even accidental dermal or inhalational exposure can result in fatal overdose, making it a significant threat to first responders and healthcare professionals. Although, carfentanil was used as veterinary medicine, the presence of it as contaminants in many illicit formulations has been reported to pose a huge risk on human health [223]. In recent years, carfentanil has emerged as a lethal adulterant in illicit opioid supplies, often mixed with heroin or counterfeit pills, contributing to waves of fatal overdoses across North America and other regions. Its extreme potency, low concentrations in biological samples, rapid pharmacokinetics, and difficulty of reversal with conventional doses of naloxone make it particularly dangerous in both clinical use and abuse and plays a key role in the escalating synthetic opioid crisis [224,225]. Further, using an in vitro cell diffusion system, the aqueous form of the drug showed better dermal permeation, indicating potential occupational risk in humans [226]. The CDC, DEA, and American College of Medical Toxicology recommend strict adherence to PPE guidelines when working with carfentanil. The recommended PPE consists of double nitrile gloves, goggles and fit-tested N95 (or higher) respirators [227]. After skin exposure, it is recommended to wash immediately with soap and water; however, one study reported that the individual continued to experience drowsiness even after decontamination with soap and water; and ultimately required administration of naltrexone [228].

While direct clinical studies remain limited, recent research highlights the potential neurobiological consequences of carfentanil exposure. A PET imaging study by Shi et al. demonstrated that MOR binding potential, measured using [11C]-carfentanil, was positively associated with cortical thickness across several brain regions in patients undergoing extended-release naltrexone treatment for opioid-use disorder [229]. These findings suggest that chronic opioid modulation of MOR signaling may contribute to structural neuroplasticity, although the role of carfentanil specifically requires further investigation. Additionally, Paterson et al. used [11C]-carfentanil PET to explore MOR availability in individuals with alcohol dependence and gambling disorder, identifying altered reward anticipation responses linked to opioid system dysregulation—potentially applicable to other forms of addiction, including carfentanil abuse [230]. Prossin et al. reported altered [11C]-carfentanil binding in limbic brain regions correlated with circulating inflammatory cytokines and mood state, implying neuroimmune interactions potentially involving astrocytes [231]. Similarly, Parker et al. highlighted [11C]-carfentanil’s application in neuroinflammation research, particularly in pain and neurodegenerative diseases, and its potential relevance for imaging glial signaling [232]. In a preclinical model, Song et al. investigated a multivalent opioid vaccine and its effect on opioid pharmacokinetics in rats co-administered a mixture of fentanyl, carfentanil, oxycodone, and heroin. The study found that the vaccine effectively reduced brain penetration of carfentanil, suggesting a potential strategy for mitigating its neurotoxic effects in polydrug scenarios [233]. Collectively, these studies underscore the urgent need to further characterize the impact of carfentanil on CNS cells, including astrocytes and neurons, particularly its influence on receptor signaling, neuroplasticity, and addiction circuitry.

## 5. Conclusions

**Figure 1:**
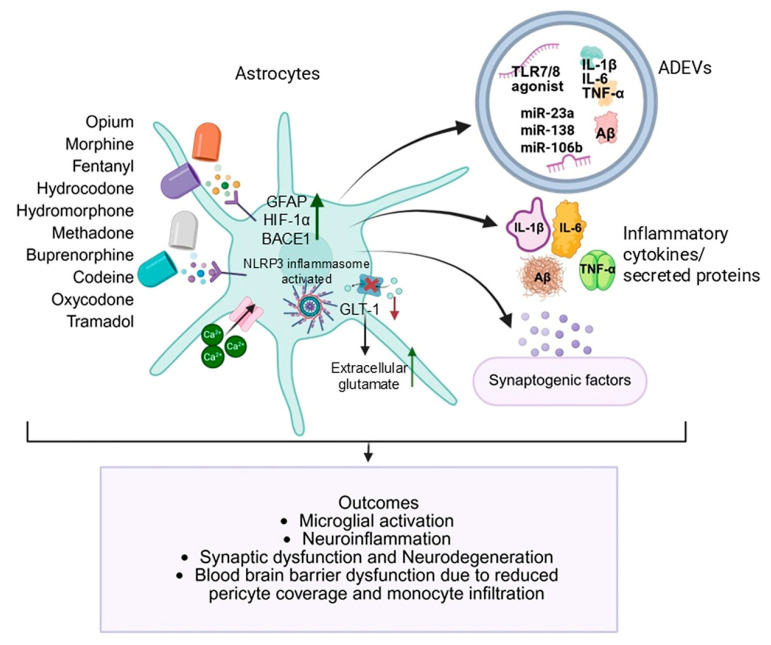
Opioid-induced astrocytic dysfunction and neuropathology. Opioids induce astrocytic activation. Activated astrocytes release ADEVs enriched in miR-23a, miR-138, miR-106b, amyloid-β, and pro-inflammatory cytokines (IL-1β, IL-6, TNF-α), along with dysregulated synaptic regulatory molecules, subsequently promoting microglial activation, neuroinflammation, synaptic dysfunction, neurodegeneration, and blood–brain barrier disruption. Green arrows indicate upregulation or activation, and the red arrows indicate downregulation or suppression. (Created in BioRender. https://BioRender.com/iwrejei, 9 September 2025).

Opioid-induced neurological disorders are multifactorial in origin, encompassing neurochemical, structural, and immunological disturbances. The emerging view of astrocytes as central players in these pathologies significantly advances our understanding of how opioids such as morphine, oxycodone, fentanyl, heroin, methadone, and others disrupt CNS homeostasis. Through both cellular reactivity and the secretion of ADEVs, astrocytes orchestrate complex intercellular signaling that influences synaptic plasticity, BBB (BBB) integrity, neuroinflammation, and neurodegeneration (Figure 1).

Recent experimental findings offer compelling evidence for the drug-specific astrocytic responses. Morphine, for instance, has been shown to trigger region-specific astrocytic amyloid deposition and neuroinflammation in non-human primates and human cell models, with ADEVs carrying amyloidogenic cargo (Aβ, APP) and inflammatory mediators like IL-1β, and regulatory RNAs including miR-138. These vesicles activate TLR7/NF-κB signaling in microglia, suppress phagocytosis, and promote BBB disruption. Notably, studies by Sil et al. and Liao et al. revealed morphine-ADEVs to be potent mediators of BBB disruption, synaptic dysfunction, and impaired microglial phagocytosis—effects that could be reversed by blocking the vesicular miRNAs or their signaling receptors [54,56]. A ~30-fold increase in AβmOC64 was observed in astrocytes of morphine-treated animals [56]. A natural derivative of morphine, codeine, though a weaker analgesic, causes oxidative stress in astrocytes, indicated by elevated lipid peroxidation, decreased antioxidant defenses (SOD, CAT), and astrogliosis in cortical and cerebellar regions [130,131]. Heroin, a potent semi-synthetic derivative, disrupts astrocytic glutamate uptake, induces hypertrophic plasticity, and alters dopamine transporter expression in astrocytes. ADEVs from heroin-exposed models carry miRNAs (e.g., let-7b, miR-206) that influence synaptic remodeling, cytokine balance, and relapse risk. Moreover, human studies show heroin modulates plasma exosomal content, including non-coding RNAs and cytokine-associated transcripts during withdrawal phases—confirming heroin’s profound neuroimmune and astrocytic consequences [138,139,140,141]. Oxycodone, a widely prescribed semi-synthetic opioid, has been linked to hippocampal astrocyte inactivation via NF-κB pathway suppression and altered exosomal signaling [150]. Astrocyte-derived biomarkers like EAAT-1 and GLT-1, proteins like NFL and α-synuclein have been found in circulation in long-term oxycodone users, reinforcing its neurodegenerative potential [151]. Interestingly, while oxycodone suppresses pro-inflammatory cytokine release in some spinal models, chronic exposure results in demyelination and neurotoxicity via astrocyte-microglia interactions and glutamate transporter dysregulation. Another semi-synthetic opioid buprenorphine, though widely used in treating opioid-use disorder, has shown concerning effects on fetal brain development. Prenatal exposure disrupts astrocyte morphology and impairs synaptogenic signaling by altering proteins such as thrombospondin-1 (TSP1) and NCAM-1. Astrocyte-derived EVs from exposed models also exhibit elevated MOR and CB1 receptor expression, suggesting disrupted neuron-glia communication and long-term neurodevelopmental risks. Hydrocodone and hydromorphone disrupt astrocytic glutamate clearance by downregulating GLT-1 and xCT and activating mGluR5, ERK, JNK, and AKT pathways—altering synaptic tone and promoting neuroimmune imbalance. Hydromorphone induces oxidative stress responses in astrocytes and modulates plasma miRNA profiles (let-7, miR-146a), which may influence tolerance and neuroimmune communication via EVs.

In the case of fentanyl, a synthetic opioid with extreme potency, its ability to induce astrocytic NLRP3 inflammasome activation and β-arrestin-mediated ERK signaling is noteworthy. These changes are associated with neuroadaptive remodeling, synaptic dysfunction, and even alterations in astrocytic calcium signaling, as seen in co-culture models and rodent cortex studies. Fentanyl’s impact on ADEV cargo, especially miR-190, suggests another layer of regulation affecting MOR expression and opioid tolerance. Methadone, a synthetic opioid used in opioid substitution therapy, demonstrates delayed glial maturation, astrogliosis, and programmed neuronal cell death, particularly in the hippocampus and cerebellum. Animal and organoid studies show significant changes in astrocyte regulatory proteins (PEA-15, p-Akt1) and elevation of TNF-α, marking methadone as a critical agent affecting neurodevelopment and adult glial dynamics.

Similarly, tramadol, through oxidative stress and activation of the PERK apoptotic pathway, leads to reactive astrocytosis and neurotoxicity—effects attenuated in some models by co-administering melatonin or elderberry extract, highlighting oxidative stress as a modifiable driver of tramadol-induced astrocytic injury. These drug-specific profiles reinforce the concept that astrocytes and ADEVs act as hubs of opioid-induced neuropathology, differentially influenced by drug type, dose, duration, and developmental context. The mechanistic insights discussed in this review are situated within the core of a growing public health crisis. OUD now affects over two million people in the U.S. alone, contributing to more than 100,000 deaths annually. The trajectory from prescription opioid misuse to illicit synthetic opioid use has compounded the challenge. Despite pharmacological diversity, a common astrocytic signature emerges across opioids: glutamate dysregulation, oxidative stress, inflammatory cytokine secretion, and vesicle-mediated intercellular toxicity. These shared astrocytic responses provide a unifying framework for understanding the neurodegenerative consequences of chronic opioid exposure.

Ongoing research efforts are actively exploring ways to counteract opioid-induced astrocyte dysfunction and its long-term neurological consequences. Several clinical trials and preclinical studies are underway focusing on modulation of glial reactivity using compounds like ibudilast, minocycline, and pioglitazone—agents known to suppress glial inflammation and restore homeostasis [234,235]. NLRP3 inflammasome inhibitors are being studied to prevent morphine-induced neuroinflammation and astrocytic activation [236]. Additionally, intranasal delivery of extracellular vesicles carrying siRNAs targeting key astrocytic pathways is being explored as a therapeutic route for reversing tolerance and restoring synaptic plasticity [123].

Future research must pivot toward dissecting the nuanced and drug-specific interactions between opioids and astrocytic EV pathways. One priority area is the temporal and spatial mapping of astrocyte dynamics and ADEVs across various brain regions under different opioid exposures. This includes understanding how acute versus chronic exposure alters the biogenesis, cargo composition, and target specificity of ADEVs. Additionally, investigations should focus on mechanistic regulators of EV release, such as Rab GTPases, ceramide pathways, and sorting complexes like ESCRT, which could be modulated to regulate EV signaling. There is also a compelling case for exploring astrocyte subtype heterogeneity—how distinct astroglial populations (e.g., A1 neurotoxic vs. A2 neuroprotective phenotypes) contribute differentially to EV-mediated neuropathology. Another essential avenue is the identification of circulating EV biomarkers, such as miR-146a-5p, miR-138, and let-7 family members, which show promise in diagnosing opioid exposure and predicting neuroinflammatory burden in both preclinical and clinical settings.

Translationally, this knowledge paves the way for glia-targeted therapies, including the delivery of engineered EVs loaded with anti-inflammatory agents or RNA-based therapeutics to reverse maladaptive opioid effects. Intranasally administered EVs, targeting MOR-silencing via siRNA cargo, and small molecules that modulate astrocyte-EV secretion represent cutting-edge strategies under investigation. Furthermore, longitudinal studies combining neuroimaging, fluid biomarkers, and cognitive assessments are essential to validate the causal links between astrocytes, ADEV signaling and long-term opioid-induced brain injury. By shifting from a neurocentric to a neuro-glial framework, future therapeutics may mitigate the neurodegenerative sequelae of opioids more precisely and with fewer side effects than traditional approaches. In summary, astrocytes and their extracellular vesicles are emerging as both biomarkers and effectors in the opioid-induced neuropathological landscape. They offer mechanistic insight into how opioids disrupt brain homeostasis and, simultaneously, therapeutic targets for intervention. Understanding and manipulating this astroglial axis holds transformative potential in addressing the dual crises of chronic pain and opioid addiction in modern medicine.

## Data Availability

No new data were created or analyzed in this study.

## Supplementary Materials

The following supporting information can be downloaded at: https://www.mdpi.com/article/10.3390/cells14181454/s1, Table S1. Classification of opioids by origin [28,211,237,238,239].
