# Supplementary material for: Astrocytes and Astrocyte-Derived Extracellular Conduits in Opiate-Mediated Neurological Disorders
Source: Cells. 2025 Sep 17;14(18):1454. doi: 10.3390/cells14181454 (PMC12468439; doi:10.3390/cells14181454)
Supplement: Supplementary file 1 [file cells-14-01454-s001.zip › cells-3844346-supplementary.pdf]

Supplementary Table 1. Classification of opioids by origin.

Group	Class	Examples	Primary Mode of Action	Use Type
Natural	Opiates (alkaloids from Papaver somniferum)	Morphine, Codeine, Thebaine, Oripavine	MOR agonists; inhibit adenylyl cyclase, suppress nociceptive transmission via (GPCR) signaling [1].	Primarily therapeutic
Semi-synthetic	Modified from natural opiates	Heroin (diacetylmorphine), Oxycodone, Hydrocodone, Hydromorphone, Oxymorphone, Buprenorphine, Nalbuphine, Dihydrocodeine, Desomorphine	Mostly MOR agonists or partial agonists; some also act on KOR or DOR; modulate pain pathways [2, 3]	Therapeutic (e.g., oxycodone), Illicit (e.g., heroin)
Synthetic	Fully synthetic opioids	Fentanyl, Sufentanil, Alfentanil, Remifentanil, Carfentanil, Methadone, Tramadol, Tapentadol, Meperidine, Levorphanol, Loperamide, Propoxyphene	Varying affinity for MOR; some inhibit serotonin/norepinephrine reuptake (e.g., tramadol, tapentadol); others block NMDA receptors (e.g., methadone) [4, 5]	Therapeutic (e.g., fentanyl), Illicit (e.g., carfentanil)

Note- MOR: Mu opioid receptor, GPCR: G-protein-coupled receptor KOR: Kappa opioid receptor, DOR: Delta opioid receptor

References

1. Stein, C., *The control of pain in peripheral tissue by opioids.* N Engl J Med, 1995. **332**(25): p. 1685-90.
2. Yaksh T, W.M., *Opioids, analgesia, and pain management*, in *Goodman & Gilman's: The Pharmacological Basis of Therapeutics, 13e*, H.-D.R. Brunton L.L., & Knollmann B.C., Editor. 2017, McGraw-Hill Education.
3. Chu Sin Chung, P. and B.L. Kieffer, *Delta opioid receptors in brain function and diseases.* Pharmacol Ther, 2013. **140**(1): p. 112-20.
4. Grond, S. and A. Sablotzki, *Clinical pharmacology of tramadol.* Clin Pharmacokinet, 2004. **43**(13): p. 879-923.
5. Herman, T.F., M. Cascella, and M.R. Muzio, *Mu Receptors*, in *StatPearls*. 2025: Treasure Island (FL).
